# The Effectiveness of the Safety and Home Injury Prevention for Seniors: A Study Protocol for a Randomized Controlled Trial

**DOI:** 10.3390/healthcare13212695

**Published:** 2025-10-24

**Authors:** Ok-Hee Cho, Hyekyung Kim, Kyung-Hye Hwang

**Affiliations:** 1 Department of Nursing, College of Nursing and Health, Kongju National University, Gongju 32588, Republic of Korea; ohcho@kongju.ac.kr; 2 Department of Nursing, Catholic Kwandong University, Gangneung 25601, Republic of Korea; hk1224@cku.ac.kr; 3 Department of Nursing, Suwon Science College, Hwaseong 18516, Republic of Korea

**Keywords:** health belief model, home accidents, safety, aged, quality of life, randomized controlled trial

## Abstract

**Background**: The majority of injuries among older adults occur due to unexpected and sudden incidents in the home environment. This study aimed to develop a protocol for the design of the health belief model-based program for preventing unintentional home injuries in older adults and to evaluate the effectiveness of the program. **Methods**: The study proposed in this protocol, Safety and Home Injury Prevention for Seniors (SHIPs), is a single-blind, parallel-group, randomized controlled trial. A total of 54 Korean older adults (≥65 years) will be randomly assigned to either (1) the intervention group (*n* = 27), which will receive the SHIPs program, or (2) the control group (*n* = 27), which will attend four lecture-only sessions. The efficacy of the program will be assessed via tests performed at baseline, 1 week after program completion, and 1 month after program completion, and analyses of the changes in injury occurrences, risk factors, preventive behaviors, beliefs about safety and injury prevention, psychological health, physiological function, and health-related quality of life. **Expected Results**: The SHIPs intervention is expected to reduce home injuries and enhance awareness and preventive behaviors among community-dwelling older adults. It may also improve their physical and psychological health and overall quality of life. **Conclusions**: The SHIPs intervention may serve as an effective community-based strategy to promote injury prevention and improve the overall well-being of older adults.

## 1. Background

Injuries among older adults are significantly associated with age-related changes in the musculoskeletal and nervous systems, underlying chronic diseases, mental health challenges, and living environments [1]. Compared to other age groups, older adults face greater exposure to risk factors owing to diminished self-protective abilities, reduced coping capacity, and delays in treatment and recovery, which increase their vulnerability to secondary injuries, disabilities, and fatalities [1,2]. These injuries significantly threaten their safety and health-related quality of life [3].

As people age, they spend more time at home, and most injuries among older adults occur unintentionally and suddenly in the home environment [4]. Preventing such unintentional home injuries requires the development and implementation of interventions that enable the recognition and management of risks in various settings. The National Council of Aging [5] recommends strategies such as fall prevention, home environment assessments, and multidisciplinary education to ensure safe daily activities, proper nutrition, medication adherence, and effective health management for older adults living at home.

Safety education for older adults should be tailored to their unique characteristics and needs. However, most safety and health programs tend to focus on school or workplace safety, offering standardized programs designed for all age groups, which fail to address the specific requirements of older adults [6]. To improve the effectiveness of safety education for seniors, it is necessary to develop standardized programs that are easy to understand, straightforward to implement, and allow for repeated practice to reinforce learning [7].

Unintentional falls are the most common type of home injury among older adults [8], with other significant causes including cuts, collisions, burns, accidents involving foreign objects or heat, and poisoning [9]. Interventions for preventing home injuries have primarily focused on fall prevention through education and exercise [10], with only a few studies addressing other frequent injury risk factors in the home environment [11]. Furthermore, home injuries among older adults result from the interplay of physical, behavioral, social, structural, and environmental factors [2,12], necessitating comprehensive, multidisciplinary prevention strategies [12].

Beliefs about home safety among older adults are shaped by their perceptions and experiences, making targeted interventions essential for fostering a sense of safety. The health belief model (HBM) provides a theoretical framework for understanding injury prevention behaviors and supporting individual-level behavior changes [13]. HBM emphasizes the crucial role of the perceptions of health and injury risks, perceived benefits of preventive actions, and personal beliefs about safety among older adults, making HBM a valuable tool for inducing positive changes in preventive behaviors [14,15]. The validity of HBM as a theoretical framework has been demonstrated in previous studies on fall prevention interventions for older adults [16,17]. Program elements in this proposed study, such as video-based lectures and peer discussion sessions, aim to influence individual beliefs and indirectly foster positive changes in safety behaviors [14,18]. In addition, exercises and hands-on practice can serve as an effective educational method by allowing participants to directly experience, practice, and acquire specific coping skills, thereby enhancing their self-efficacy [19].

This study aimed to develop a protocol for the design of an HBM-based program to prevent unintentional home injuries in older adults. The program focuses on six key areas of home injury prevention and management: falls, cuts and collisions, burns and fires, swallowing disorders, medication management, and dehydration/heat-related illnesses. The program, termed “Safety and Home Injury Prevention Program for Seniors” (SHIPs), is designed to motivate older adults to adopt home safety and prevention behaviors, empower older adults to identify risk factors, and implement preventive actions, ultimately reducing injury occurrences. In addition, the program is designed to enhance psychological health, physical function, and quality of life, all of which may be adversely affected by injury incidents [20]. Furthermore, the protocol aimed to assess the feasibility of implementing the program before pursuing additional funding for a randomized controlled trial. The findings of this study are expected to provide practical guidelines for educators involved in injury prevention initiatives.

### 1.1. Aims

#### 1.1.1. Primary Objectives

To evaluate the effectiveness of a Health Belief Model (HBM)-based health education program on the number of unintentional home injuries among community-dwelling older adults from baseline to post-intervention and at one-month follow-up.

#### 1.1.2. Secondary Objectives

- To assess changes in the Health Belief Model (HBM) constructs—including home injury risk assessment, injury prevention behaviors, and beliefs about safety and injury prevention—among community-dwelling older adults from baseline to post-intervention and at one-month follow-up.
- To examine the effects of the program on psychological health (depression, fear of falling), physiological function (muscle strength, timed up and go test, one-leg standing test), and health-related quality of life among community-dwelling older adults, measured at baseline, immediately after the intervention, and one month after the intervention.

### 1.2. Research Hypothesis

Participants receiving the Health Belief Model (HBM)–based health education program are expected to show a significantly greater reduction in the number of unintentional home injuries from baseline to post-intervention and at one-month follow-up compared with those receiving usual care.

## 2. Methods

### 2.1. Study Design

**Figure 1:**
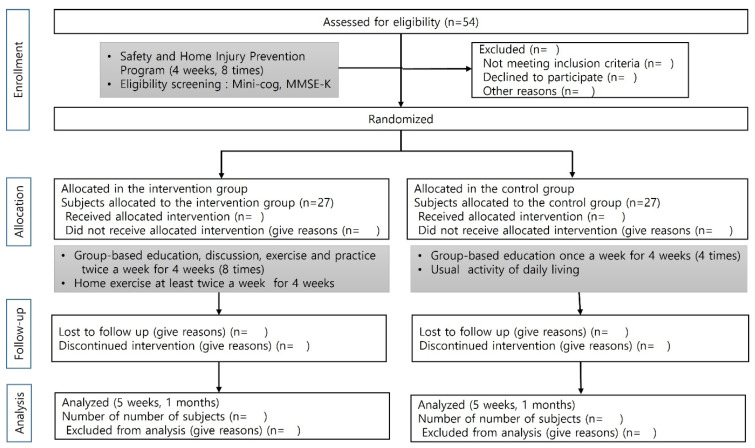
Flow diagram of study design.

This protocol adheres to the guidelines outlined in the Standard Protocol Items: Recommendations for Interventional Trials (SPIRIT) [21]. The study for the efficacy analysis of the SHIPs will use a single-blinded (assessor), parallel-group, randomized controlled trial design. The protocol was registered with the Clinical Research Information Service (https://cris.nih.go.kr, accessed on 15 July 2025) (Figure 1).

### 2.2. Sampling/Participants

To recruit participants, the research objectives and eligibility criteria will be presented to administrators of senior welfare centers in the Chungcheong region of South Korea for their cooperation. Participants who agree to take part and sign the informed consent form will be included in the study. Participants will be recruited through recruitment notices posted on bulletin boards and in the community, as well as through recommendations from welfare center staff, and individuals who meet the following inclusion criteria will be invited to participate: (1) aged 65 years or older; (2) not currently receiving treatment for fractures or acute illnesses; (3) classified as “robust” or “pre-frail” (K-FRAIL ≤ 2); (4) capable of exercising independently; (5) classified as “non-dementia” based on the Mini-Cog test; (6) can communicate and cooperate during anthropometric assessments; and (7) no participation in similar programs within the past 6 months.

Eligibility screening for inclusion criteria 3 and 5 will involve administering the K- K-FRAIL scale and the Mini-Cog test. The K-FRAIL scale (Korean version of the FRAIL scale) [22] evaluates frailty levels using a simple five-item questionnaire (fatigue, resistance, ambulation, illness, and weight loss) that does not require a physical examination. Each item is rated 0 or 1 point, and total scores are interpreted as follows: 0 = robust, 1–2 = pre-frail, and 3–5 = frail. The Mini-Cog test [23] assesses cognitive function through a three-step process: three-word registration, clock drawing, and three-word recall. Participants who recall all three words or recall 1–2 words while correctly drawing the clock are classified as “non-dementia.” Those who recall 1–2 words but fail to draw the clock correctly, and those who fail to recall any words are classified as having a “positive screen” for dementia. This test is widely recognized for its simplicity and reliability in screening for cognitive impairment.

The exclusion criteria will include: individuals with pain, musculoskeletal disorders, or neurological conditions that impair balance.

### 2.3. Sample Size

Sample size calculation was performed using G*Power 3.1.9.2. (Heinrich-Heine-University, Düsseldorf, Germany) Assuming a medium effect size of 0.25 [24], an alpha level of 0.05, a target statistical power of 0.80, an intraclass correlation coefficient of 0.5 to account for the correlation between repeated measures, two groups, and three measurement timepoints, the minimum required sample size was calculated to be 44 participants (22 per group). To accommodate a potential 20% dropout rate during the intervention period, the study aims to recruit a total of 54 participants, with 27 allocated to the intervention group and 27 to the control group.

### 2.4. Recruitment

After posting recruitment notices, potential participants who express interest will be contacted via phone to confirm their willingness to participate. Information sheets will be provided either in person or via email, allowing individuals sufficient time to review the study details. The information sheet will outline the study’s purpose, program, benefits, potential risks, the right to withdraw voluntarily at any time, and assurances regarding the confidentiality of personal information. Participants will be officially registered upon submitting a signed informed consent form to the research team.

### 2.5. Randomization

Participants will be randomly assigned to (1) the intervention group, which will receive the SHIPs program comprising eight sessions of lectures, discussions, and experiential activities, or (2) the control group, which will receive four lecture-only sessions. Block randomization will be performed with a 1:1 allocation ratio (intervention: control). A statistician will input the IDs of participants who have provided informed consent into a Microsoft Excel 2016 datasheet and generate unique allocation codes to ensure concealment. Randomization sequences with mixed block sizes of four and six will then be produced using SAS version 9.2 (SAS Institute Inc., Cary, NC, USA).

Sealed envelopes containing allocation codes and names of study participants will be prepared by a statistician and placed in an opaque envelope, which will be provided to the research team. To ensure allocation concealment, a separate evaluator will open one sealed, sequentially numbered opaque envelope per participant, in the order of their arrival, to determine group assignment. The randomization list will be securely kept by the statistician, ensuring that intervention researchers remain blinded to group assignments. To further maintain blinding, separate evaluators will be assigned to assess the participants, and neither the participants nor the evaluators will be aware of the group assignments. Standardized scripts were used during recruitment and data collection to ensure consistent communication, and the intervention and control sessions were conducted separately at different sites to prevent potential information exchange or cross-group influence.

### 2.6. Development of the SHIPs Intervention

#### 2.6.1. Aims and Objectives

SHIPs aims to empower older adults to protect themselves from injury by fostering beliefs about safety and prevention while promoting preventive behavior habits. The program focuses on four important areas: HBM-based program design, promotion of actionable behavioral change, strategies for injury prevention and management, and development of standardized educational materials and manuals for injury prevention.

The program’s structure is based on the HBM [14,18], designed to motivate individuals to adopt health-promoting behaviors through four key constructs: perceived susceptibility, severity, benefits, and barriers. By incorporating these HBM concepts, the program aims to encourage participants to engage in preventive behaviors. First, it improves safety awareness and highlights participants’ vulnerability to risks (perceived susceptibility: risk factor assessment). Second, it motivates action by emphasizing the potential consequences of inaction (perceived severity: consequences of injuries and their effects on quality of life). Third, it emphasizes the advantages of taking preventive measures (perceived benefits: effectiveness of preventive activities). Finally, it addresses and mitigates obstacles (perceived barriers: barrier reduction and coping strategies) to increase the likelihood of behavior change. This program incorporates self-efficacy as an additional key construct alongside the four core HBM constructs, emphasizing the crucial role of self-belief in enabling participants to sustain preventive behaviors over the long term.

#### 2.6.2. Educational Content and Teaching Methods

The program curriculum was developed based on literature reviews and statistical data [15,25,26,27,28,29,30,31,32,33], prioritizing six important areas of prevention and management at home: fall prevention, cut and collision prevention, burn and fire prevention, swallowing disorder management, medication management, and dehydration/heat-related illness prevention. The teaching methodology was designed to account for the cognitive and social characteristics of older adults, maximizing educational effectiveness by combining lecture-based and discussion-based approaches with demonstrations and hands-on practice.

The program consists of eight sessions, each lasting 60–70 min, divided into four lecture and discussion sessions and four experiential activity sessions [19,34]. The specific details of each session are outlined in Table 1. Lecture-based teaching methods were employed to systematically and effectively deliver a large volume of educational content to multiple participants in a short period [35].

During the lecture sessions (1st, 3rd, 5th, and 7th sessions), participants first watch case-based animated videos illustrating injury mechanisms in the six focus areas, providing foundational knowledge about home safety and encouraging preventive behaviors. After the videos, presentations are used to expand on topics such as injury risk factors and coping strategies. Each lecture concludes with participants completing picture quizzes and receiving small rewards to reinforce key messages, boost motivation, and enhance learning outcomes.

In the discussion sessions (3rd, 5th, and 7th sessions), participants share their experiences with implementing preventive actions to avoid injuries. Real-life testimonials from peers are incorporated to foster self-efficacy and promote the exchange of ideas. While instructor involvement is kept to a minimum, corrective guidance on preventive behaviors is provided through questions and answers to ensure meaningful and productive discussions.

The program incorporates exercises and hands-on activities to provide participants with opportunities to learn and practice home safety, injury prevention behaviors, and coping strategies. To promote practical behavioral change, experiential activities are included in the 2nd, 4th, 6th, and 8th sessions. These sessions feature fall prevention exercises (2nd session), wound disinfection and hand hygiene practice (4th session), swallowing disorder prevention exercises and first aid training, including the Heimlich maneuver (6th session), and activities focused on proper hydration and medication management (8th session). These activities are designed to help participants apply preventive behaviors effectively in their daily lives.

The fall prevention exercises were adapted from a previous study [29] and structured into beginner and intermediate levels to encourage older adults at risk of frailty or with mild mobility restrictions. The exercises, which can be performed daily at home, include functional and chair-based exercises such as marching in place, walking between rooms, wall push-ups, leg lifting and tapping, seated walking, standing up from a chair, arm stretching, calf stretches, and hamstring stretches. Each session includes group exercises, and participants are encouraged to continue these exercises individually at home at least twice a week. A previous study [36] reported that these exercises improve mobility, walking scores, and balance confidence. Participants are asked to track their home exercises and any unintentional injuries on a sticker chart, which they submit during group training sessions for monitoring and feedback purposes.

#### 2.6.3. Development of Educational Materials and Program Validation

The educational materials were developed separately for instructors and participants (tailored to older adults). The teaching materials include standardized teaching guides detailing learning objectives, lecture tips, and important points, along with PowerPoint slides, a manual, weekly handouts, and activity sheets. The learning materials were designed to align with the particularities of older adults, using large fonts, accent colors, and illustrations to improve readability and comprehension. To reinforce learning, videos were created for each of the six injury areas, covering case studies, educational content, and experiential activities.

The completed program and materials were validated by an expert panel consisting of four physicians (specialists in emergency medicine, orthopedics, neurosurgery, and rehabilitation medicine), two professors of geriatric nursing, one exercise specialist, one occupational therapist, and one physiotherapist. After explaining the program’s objectives, the panel evaluated the content, methodology, and feasibility of on-site application. With all items scoring 4 or higher out of 5, the program’s validity was established.

### 2.7. Procedures

Participants who provide consent will undergo eligibility screening, including general characteristics (age, sex, comorbidities) and the K-FRAIL and Mini-Cog tests. Thereafter, eligible participants will be assessed at baseline for demographic characteristics, number of unintentional home injuries over the past 6 months, risk factors, preventive activities, beliefs, psychological health, physical function (grip strength, timed up-and-go test, and one-leg standing test), and health-related quality of life. Randomization will be performed after the baseline assessments. The intervention will span 4 weeks, with the planned programs delivered to the intervention and control groups. Although the frequency of sessions differed between groups (twice per week for the intervention group and once per week for the control group), the total intervention period was identical (4 weeks). The higher session frequency in the intervention group reflects the inclusion of both educational and behavioral components, which required additional sessions for practice and feedback. The control group received lecture-based education only. To minimize potential attention bias associated with different contact times, both groups received comparable educational content, and follow-up monitoring was implemented at similar intervals throughout the study period. Post-intervention assessments will be conducted 1 week and 1 month after program completion at a designated location.

#### 2.7.1. The Intervention Group

Participants in the intervention group will participate in the SHIPs intervention, which integrates lectures, discussions, and experiential activities over 4 weeks. The program consists of eight sessions (two per week), each lasting 60–70 min. The sessions will be conducted in accessible locations, such as senior welfare center classrooms, with small groups of approximately 10 participants. Lectures and discussions will be led by the researcher, and experiential activities will be facilitated with support from a research assistant.

Program adherence will be evaluated by tracking attendance, compliance with home exercises, results from functional ability tests, and the number and percentage (95% confidence interval) of participants who complete the questionnaire assessments. Attendance compliance will be defined as attending 80% or more of the weekly group education and exercise sessions and participating in at least five sessions.

#### 2.7.2. The Control Group

The control group will receive lecture-based education, using the same booklets, videos, and picture quizzes as the intervention group but without the inclusion of discussion components. Sessions will be held in senior welfare center classrooms, with no restriction on the number of participants per group. The lectures will be delivered by the researcher. Pre- and post-assessment methods and procedures will be the same as those used in the intervention group.

### 2.8. Outcomes Measures

#### 2.8.1. Primary Outcome Variables

The number of unintentional home injuries, including falls, cuts/collisions, burns/fires, medication-related incidents, swallowing disorders, and dehydration/heat-related illnesses, will be recorded. At baseline, the number of incidents occurring over the past 6 months will be assessed, whereas at 1 week and 1 month after the intervention, the number of incidents from the preceding month will be evaluated.

#### 2.8.2. Secondary Outcome Variables

##### Home Injury Risk Assessment

Home injury risk will be assessed using a scale developed by the researcher, based on expert consultation and prior research [25,26,27,28,30,31,37,38,39]. The scale comprises 79 items categorized into five subscales: environmental risk assessment (37 items covering areas such as entrances, hallways, living rooms, bedrooms, and kitchens), fall injuries (12 items), cut and collision injuries (12 items), medication management (8 items), and swallowing disorders (10 items). Responses are scored on a binary scale (Yes = 1, No = 0), with higher total scores indicating a greater number of identified injury risk factors.

##### Home Injury Prevention Behaviors

Injury prevention behaviors at home will be assessed using a scale developed by the researcher, based on expert input and previous studies [25,26,27,28,30,31,32,38,40]. The 71-item scale is divided into six subscales: fall 13 items), cut and collision (7 items), burn and fire (24 items), medication management behaviors (7 items), swallowing disorder (9 items), and dehydration and heat-related illness prevention behaviors (9 items). Responses are scored on a binary scale (Yes = 1, No = 0), with higher total scores reflecting greater compliance with injury prevention behaviors.

The scale’s validity was confirmed by an expert panel comprising five professionals specializing in geriatrics, preventive medicine, geriatric nursing, emergency medical services, and occupational therapy. The item-content validity index (I-CVI) exceeded 0.80, and the scale-content validity index (S-CVI/Ave) was 0.90. The reliability of the scale, measured using Cronbach’s ⍺, was 0.87 in a pilot survey. The instruments were designed as self-administered questionnaires for community-dwelling older adults, and all assessors received standardized training prior to data collection to ensure consistency in administration and participant guidance.

##### Beliefs About Safety and Injury Prevention Behaviors

Beliefs about home safety and injury prevention behaviors will be measured using a scale developed by the researcher, based on the HBM [14,15,18] and previous studies [16,41,42]. The scale comprises five subscales: perceived susceptibility (four items), perceived severity (three items), perceived benefits (three items), perceived barriers (six items), and self-efficacy (four items). Each item will be rated on a 5-point Likert scale (1 = strongly disagree, 5 = strongly agree), with higher scores indicating stronger beliefs in preventive behaviors. The Cronbach’s ⍺ of this scale was 0.92 in a pilot survey.

##### Psychological Health

- Depression

Depression will be assessed using the Korean version of the Geriatric Depression Scale-Short Form (GDS-SF) [43]. This 15-item scale utilizes a dichotomous response format, with “Yes” scored as 1 point and “No” as 0 points. Total scores range from 0 to 15, with higher scores indicating greater depressive symptomatology. Scores are categorized as follows: 0–5: no depression; 6–10: mild depression; 11–15: severe depression. In a previous study [43], the GDS-SF demonstrated a good internal consistency, with a Cronbach’s ⍺ of 0.88.

- Fear of Falling

The fear of falling will be assessed using the Korean version of the Falls Efficacy Scale-International (FES-I) [44]. This 16-item scale utilizes a 4-point Likert scale (1 = Not at all concerned, 4 = Very concerned). Total scores range from 16 to 64, with higher scores indicating greater fear of falling. A score of 23 or higher is considered indicative of significant fear of falling [45]. A previous study [44] reported an excellent internal consistency for the FES-I, with a Cronbach’s ⍺ of 0.96.

##### Physiological Function

- Muscle Strength: Grip Strength

Grip strength will be measured using a hand dynamometer (DongHwa Science Skill Takei, TKK-5401, Seoul, Republic of Korea). Participants will perform two trials with each hand. During each trial, they will hold the dynamometer with their arms lowered at their sides, angled outward at approximately 30 degrees, with the back of the hand facing outward, and exert maximum force. If testing both hands is not possible, the available measurement will be used. Higher dynamometer readings indicate greater grip strength. Grip strength is highly correlated with overall muscle strength, and it is widely regarded as a safe and reliable method for assessing strength in older adults [46].

- Timed up and go test

To assess dynamic balance, the timed up and go test will be administered [47]. In this test, participants begin seated in a chair and, upon the tester’s signal, stand up, walk 3 m, turn around, walk back to the chair, and sit down again. The time taken to complete the sequence is recorded. A time of 30 s or more is generally considered indicative of an increased risk of falls and may suggest the need for assistance with activities such as walking, climbing stairs, or going outdoors. Participants will perform the timed up-and-go test three times, and the mean value will be used as the final result.

- One-leg standing test

Static balance will be assessed using the one-leg standing test [48]. In the one-leg standing test, participants stand on a flat surface with their arms at their sides and their eyes open. They are instructed to select a comfortable leg to stand on while lifting the opposite foot off the ground. The time is measured from the moment they lift their foot until the lifted foot touches the floor. The longer of the two measurements is recorded as the final score. Higher scores indicate better static balance.

##### Health-Related Quality of Life

The health-related quality of life will be assessed using the Korean version of the 12-item Short Form Health Survey (SF-12) [49]. The SF-12 questionnaire comprises 12 items that evaluate physical and mental health across eight domains: physical functioning, physical role limitations, pain, general health perceptions, vitality, social functioning, emotional role limitations, and mental health. Each item is rated on a 3- or 5-point scale, with higher scores indicating better quality of life. In a previous study [50], the tool demonstrated an excellent internal consistency, with a Cronbach’s ⍺ of 0.91.

### 2.9. Data Analysis

Data analysis will be performed using SPSS version 26 (IBM Corp., Armonk, NY, USA). Descriptive statistics, including means, medians, frequencies, and percentages, will be used to analyze the general characteristics of the intervention and control groups. Normality of continuous variables will be assessed using the Shapiro–Wilk test. For normally distributed data, the homogeneity of participant characteristics and variables between groups prior to the intervention will be tested using the χ^2^ test, unpaired *t*-test, and one-way analysis of variance. Differences in score changes between the intervention and control groups at the three timepoints—baseline (T0), 1 week after intervention (T1), and 1 month after intervention (T2)—will be analyzed using repeated measures analysis of variance to evaluate between-group differences, within-group differences across the three timepoints, and interactions. For non-normally distributed data, analyses will be conducted using the Mann–Whitney U test and generalized estimating equations. All analyses will follow the intention-to-treat (ITT) principle, including all randomized participants in their originally assigned groups, regardless of adherence or withdrawal. Missing data will be handled using multiple imputation based on the fully conditional specification (FCS) method within SPSS version 26.0. Covariates such as age, sex, and baseline scores will be included to control for potential confounding factors.

To ensure robust statistical evaluation, Type I (alpha) error control will be prioritized, and the Holm–Bonferroni correction will be applied to maintain the overall significance level at *p* < 0.05 (two-tailed). An interim analysis will be descriptive only, focusing on data completeness and participant safety, without formal hypothesis testing to avoid Type I error inflation. Statistical significance will be set at a *p*-value of 0.05. Care will be taken to ensure that all participants complete the program and data collection, minimizing missing data through active follow-up.

### 2.10. Ethical Consideration

This study was approved by the Institutional Review Board (IRB) of Kongju National University, granted exemption status on 22 July 2024 (No. KNU_2024-07). This study design complies with the Declaration of Helsinki. Before data collection, all study participants will receive information about the study’s purpose, procedures, data collection period, their right to withdraw at any time without penalty, and that their data will be confidential and will be stored in a safe place. All participants will be invited to read an informed consensus and will be assured. Informed consent will be obtained from participants before proceeding with data collection.

All data collected from participants during the study will be treated with utmost confidentiality and privacy, ensuring participant anonymity through the use of unique identifiers; data will be collected only after obtaining prior informed consent. Participants will be guaranteed that study results will be published in an aggregated form, not allowing the identification of participants. Research assistants will be trained to respect participants’ status and time during data collection. If they notice participants becoming tired during data collection, they will offer a pause. If the participant exhibits excessive emotional burden, we will provide tailored interventions and assess whether the participant can continue the program. If the outcome is negative, the data collected up to that critical point will be retained, and the reasons for study withdrawal will be documented accordingly.

Participant data will initially be recorded in a research notebook and subsequently transferred to a secure storage device. All information will be securely stored in a separate, designated location under the supervision of the principal investigator. Collected data will be retained for 5 years after the study’s completion and will be permanently deleted afterward. Electronic files will be password-protected, with the passwords regularly updated to maintain security. Access to personal data will be strictly limited to the principal investigator, co-researchers, evaluators, data managers, and authorized members of the research team.

### 2.11. Data Monitoring

An interim analysis of the measurement data will be conducted 1 week after the intervention to evaluate the effectiveness, validity, and safety of the intervention. To ensure objectivity and minimize bias, this analysis will be performed by an external statistician. If any potential safety concerns are identified during the analysis, the statistician will immediately report the findings to the principal investigator. Thereafter, the principal investigator will carefully review the interim analysis results and make an informed decision on terminating or continuing the study.

### 2.12. Harms

All study participants will be continuously monitored for the occurrence of adverse events (AEs) and serious adverse events (SAEs) throughout the intervention period and the one-month follow-up. An adverse event (AE) is defined as any undesirable or unintended medical occurrence experienced by a participant, regardless of its relationship to the intervention. A serious adverse event (SAE) refers to any event that results in death, is life-threatening, requires hospitalization, or causes persistent disability.

The safety of participants is the top priority of this study, and a systematic approach will be applied to both the implementation and evaluation of the program. Data will be collected at three timepoints: before the intervention, one week after the intervention, and one month after the intervention. All reported events will be systematically recorded in a standardized Excel file. In the event that an unintentional home injury occurs during the study period, participants will be instructed to contact the research team immediately. If a change in a participant’s clinical condition is identified during the intervention or follow-up period that meets the criteria for discontinuation, participation will be terminated immediately to ensure safety. In such cases, participants will be referred for primary care or, if necessary, specialized medical evaluation and follow-up. The research team will conduct additional follow-up to confirm the participant’s recovery and safety status.

### 2.13. Patient and Public Involvement

An expert panel participated in the development, evaluation, and refinement of the injury prevention program. Facility managers at senior welfare centers will assist with participant recruitment, measurements, and program operation planning.

### 2.14. Expected Results

The SHIPs intervention is expected to produce measurable positive improvements among community-dwelling older adults. Following the intervention, participants are anticipated to experience a reduction in the number of home injuries, an increase in awareness of injury risks, and greater engagement in preventive behaviors. Their beliefs about home safety and injury prevention are also expected to be strengthened. Furthermore, the intervention is expected to contribute to improvements in physical functioning, psychological health, and overall health-related quality of life. Continuous support and monitoring throughout the intervention period are expected to promote program adherence, verify its effectiveness, and minimize the risk of unplanned dropouts or accidents. These expected outcomes align with the theoretical framework of this study and are supported by previous evidence suggesting that injury-prevention education interventions for older adults can yield synergistic benefits [22,34].

## 3. Trial Status

The study is scheduled to begin on 1 April 2025 and conclude on 31 January 2026. The timeline for the participants is presented in Table 2.

## 4. Discussion

This protocol seeks to address this broader scope by identifying the causes of major unintentional injuries, including falls, and integrating these insights into prevention programs to establish an evidence-based approach.

A major strength of this proposed study is its integrated approach to different injury types. The injury categories were selected based on previous research and national injury statistics reports [8,12,51], prioritizing six high-frequency injury areas in the home. By incorporating home environment risk factor assessments and delivering diverse activities and educational materials specifically tailored to the needs of older adults, an integrated multidisciplinary safety program was developed.

The significance of this program lies in its practical educational design, which simplifies complex safety behaviors into key actionable components, making them feasible to implement. Furthermore, it incorporates experiential activities to facilitate skill acquisition. It is anticipated that future implementation and evaluation of the program will provide valuable feedback for further refinement and enhancement.

Falls are the most common cause of injuries among older adults, and the proposed study aims to address them as a major focus, incorporating evidence-based interventions. Previous studies have demonstrated that exercise is an effective strategy for fall prevention in community-dwelling older adults [52], with a particular emphasis on balance and functional training [53]. In line with these findings, this study will adopt a home-based exercise program developed by Jones & Frederick [29]. The program features simple, easy-to-perform exercises that require no special equipment and are tailored to the physical characteristics of older adults. In this study, group exercises will be conducted during each session, and participants will be instructed to perform two additional individual exercise sessions at home each week.

When designing programs for older adults, it is important to consider their learning abilities. This program was developed based on recommendations for health education for older adults [54], incorporating the preferences, physical abilities, and cognitive capacities of older adults. It includes a variety of formats, such as lectures, discussions, question-and-answer sessions, demonstrations, videos, and experiential activities. To enhance comprehension, booklets and educational videos were specifically developed and utilized. These materials were designed with consideration of the literacy levels of older adults, informed by literature reviews and expert consultations. They include animations and real-life case videos to increase engagement and immersion. In addition, the program incorporates experiential activities to enable participants to apply injury prevention education at home.

The program is designed to be implemented with the cooperation of senior welfare centers and to support its sustained use by senior organizations. If proven effective, the program can be expanded to other regions or organizations, facilitating long-term evaluations of its effect and cost-effectiveness. Expanding the program’s application to broader populations would help achieve universal health management and ultimately contribute to reducing societal costs associated with unintentional injuries among older adults.

Furthermore, this program curriculum was developed based on the HBM [13,14,15], a well-established framework for designing preventive behavior interventions. Perceived susceptibility, severity, benefits, and barriers associated with each injury type were addressed using tools such as risk factor checklists, case videos, lectures, discussions, and prevention behavior checklists. To further improve preventive behaviors, which is the primary goal of the program, picture quizzes and experiential activities were incorporated to foster self-efficacy. By addressing these important HBM constructs and enhancing self-efficacy, the program aims to improve participants’ knowledge about safety incidents, encourage the adoption of preventive behaviors, and ultimately reduce the occurrence of injuries.

Safety issues and risks affect all aspects of life, and excessive sensitivity to these risks can lead to overcompensation, unnecessary expenses, fear, and anxiety, ultimately reducing the quality of life [7,55]. In particular, excessive fear of falls—a major safety concern for older adults—can limit physical activity, resulting in declines in physical and cognitive function and reduced quality of life [55]. The proposed study aims to address these challenges by promoting appropriate safety awareness and providing effective coping strategies to reduce unnecessary fear, encourage physical activity, and achieve positive outcomes.

This study faces several challenges. This study has several limitations. First, it was conducted in a single region and included a relatively small sample of older adults in the pre-frailty stage, which may limit the generalizability of the findings to other regions or populations. Second, maintaining participant engagement throughout the eight-session program may be challenging, and there is a risk of attrition during the 1-month follow-up period. Consistent adherence to the program curriculum is essential for accurately evaluating its outcomes, underscoring the importance of fostering strong researcher-participant relationships [56]. To encourage sustained participation, the program will consider participants’ preferred times and locations, provide incentives, offer personalized feedback on the program’s benefits, and maintain regular communication through short message service reminders. Third, participants may perceive the various tools and assessments, which are essential for evaluating multiple outcomes, as burdensome. To address these concerns, trained evaluators will conduct individualized, accurate, and efficient assessments to minimize inconvenience for participants. For the primary outcome measure—the number of injury incidents—participants will be encouraged to maintain injury logs to reduce the risk of recall bias. These logs will be regularly reviewed and reinforced through researcher intervention to ensure completeness and accuracy. In addition, the relatively short follow-up period restricts the ability to evaluate the long-term effects of the intervention; therefore, future studies should include extended follow-up assessments to confirm the sustainability of the observed outcomes.

## 5. Conclusions

This protocol aims to support education to prevent unintentional injuries in older adults at home, focusing on six home injury domains selected through a high-frequency integrated risk factor assessment tailored to the home environment. Safety and home injury prevention education, based on the Health Belief Model, aims to foster beliefs about safety and prevention and cultivate preventive behavior habits so that older adults can protect themselves from injury.

Furthermore, the program simplifies complex safety behaviors into actionable components and promotes skill acquisition through experiential learning, enabling practical and realistic education design. Because this study addresses not only home safety accidents but also clinical aspects such as swallowing disorders and medication management, it has the potential to improve self-efficacy and quality of life in older adults. Furthermore, promoting confident safety and home injury prevention behaviors will contribute to reducing preventable falls. These findings will provide evidence to support a scalable and sustainable safety and home injury prevention education model that applies the health belief model.

## Figures and Tables

**Table 1:** Composition of the safety and home injury prevention program.

Section	Week		Key Concept	Activity
Fall prevention at home	1	Perceived Susceptibility	Risk factors for falls	Risk factor checklist Watching case-based videos Lecture (video, summary)
		Perceived Severity	Consequences of falls and their impact on quality of life	
		Benefits	Effectiveness of preventive activities	Sharing injury and prevention experiences Preventive activity checklist
		Barriers	Overcoming barriers to falls and coping strategies	
	2	Self-Efficacy	Identifying appropriate preventive actions Home-based fall prevention exercises (beginner level)	Picture quiz Exercise (video, demonstration, workout)
Cut and collision prevention at home	3	Perceived Susceptibility	Risk factors for cuts and collisions	Risk factor checklist Watching case-based videos Lecture (video, summary)
		Perceived Severity	Consequences of cuts and collisions	
		Benefits	Effectiveness of preventive activities	Sharing injury and prevention experiences Preventive activity checklist
		Barriers	Overcoming barriers to cuts and collisions and coping strategies	
		Self-Efficacy	Identifying appropriate preventive actions	Picture quiz
Burn and fire prevention at home	4	Perceived Susceptibility	Risk factors for burns and fires	Risk factor checklist Watching case-based videos Lecture (video, summary)
		Perceived Severity	Consequences of burns and fires	
		Benefits	Effectiveness of preventive activities	Sharing injury and prevention experiences Preventive activity checklist
		Barriers	Overcoming barriers to burns and fires and coping strategies	
		Self-Efficacy	Wound disinfection management	Wound disinfection (video, demonstration, hands-on practice)
Swallowing disorder management at home	5	Perceived Susceptibility	Risk factors for swallowing disorders	Risk factor checklist Watching case-based videos Lecture (video, summary)
		Perceived Severity	Consequences of swallowing disorders and their impact on quality of life	
		Benefits	Effectiveness of self-management activities	Sharing injury and prevention experiences Preventive activity checklist
		Barriers	Overcoming barriers to swallowing disorder management and coping strategies	
	6	Self-Efficacy	Identifying appropriate self-management activities Swallowing disorder prevention exercises	Picture quiz Exercise (video, demonstration, workout), First aid (video, demonstration, hands-on practice)
Medication management at home	7	Perceived Susceptibility	Risk factors for medication misuse and addiction	Risk factor checklist Watching case-based videos Lecture (video, summary)
		Perceived Severity	Consequences of medication misuse and addiction	
		Benefits	Effectiveness of self-management activities	Sharing injury and prevention experiences Preventive activity checklist
		Barriers	Overcoming barriers to medication management and coping strategies	
		Self-Efficacy	Identifying appropriate self-management activities Medication storage and measurement methods	Picture quiz Medication storage and measurement methods (video, demonstration, hands-on practice)
Dehydration and heat-related illness prevention at home	8	Perceived Susceptibility	Risk factors for dehydration and heat-related illness	Risk factor checklist Watching case-based videos Lecture (video, summary)
		Perceived Severity	Consequences of dehydration and heat-related illness	
		Benefits	Effectiveness of self-management activities	Sharing injury and prevention experiences, Preventive activity checklist
		Barriers	Overcoming barriers to dehydration management and coping strategies	
		Self-Efficacy	Identifying appropriate preventive activities Healthy water drinking	Picture quiz Healthy water drinking (video, demonstration, hands-on practice)

**Table 2:** Schedule of safety and home injury prevention program.

Study Protocol					Closing
	Enrollment	Allocation	Intervention (4 Weeks)	Post- Allocation (5 Weeks)	Post- Allocation (1 Month)
Timeline	Tx	T0	-	T1	T2
Enrollment					
Eligibility screening	X				
Informed consent	X				
Allocation		X			
Interventions: Safety and home injury prevention program					
Intervention group (twice a week)		X	X	X	X
Control group (once a week)		X	X	X	X
Assessments					
Mini-cog test		X			
K-FRAIL scale		X			
Age/sex		X			
Acute illnesses		X			
Fracture		X			
ADL		X			
Balance impairment		X			
Cerebrovascular disease		X			
Active severe pain		X			
Participate in other exercises		X			
Measures					
Number of unintentional home injuries		X	X	X	X
Home injury risk assessment		X	X	X	X
Home injury prevention behaviors		X		X	X
Beliefs about safety and injury prevention behaviors		X		X	X
Psychological health					
Depression		X		X	X
Fear of falling		X		X	X
Physiological function					
Grip strength		X		X	X
Time up and go test		X		X	X
One-leg standing test		X		X	X
Health-related quality of life		X		X	X

Note: Tx = enrollment; T0 = baseline; T1 = 1week after intervention; T2 = 1 month after intervention; K-FRAIL scale = Korean version of the FRAIL scale; ADL = Activities of Daily Living.

## Data Availability

No datasets were generated or analyzed during the current study, as this is a study protocol. Therefore, data sharing is not applicable.

## Abbreviations

SHIPs	Safety and Home Injury Prevention for Seniors
HBM	Health belief model
SPIRIT	Standard Protocol Items: Recommendations for Interventional Trials
GDS-SF	Geriatric Depression Scale-Short Form
FES-I	Falls Efficacy Scale-International
SF-12	12-Item Short Form Health Survey
